# Single-Cell Hypertrophy Promotes Contractile Function of Cultured Human Airway Smooth Muscle Cells via Piezo1 and YAP Auto-Regulation

**DOI:** 10.3390/cells13201697

**Published:** 2024-10-14

**Authors:** Kai Ni, Bo Che, Rong Gu, Chunhong Wang, Yan Pan, Jingjing Li, Lei Liu, Mingzhi Luo, Linhong Deng

**Affiliations:** Changzhou Key Laboratory of Respiratory Medical Engineering, Institute of Biomedical Engineering and Health Sciences, School of Medical and Health Engineering, Changzhou University, Changzhou 213164, China

**Keywords:** ASMC hypertrophy, contractile function, cell volume, membrane tension, Piezo1, YAP

## Abstract

Severe asthma is characterized by increased cell volume (hypertrophy) and enhanced contractile function (hyperresponsiveness) of the airway smooth muscle cells (ASMCs). The causative relationship and underlying regulatory mechanisms between them, however, have remained unclear. Here, we manipulated the single-cell volume of in vitro cultured human ASMCs to increase from 2.7 to 5.2 and 8.2 × 10^3^ μm^3^ as a simulated ASMC hypertrophy by culturing the cells on micropatterned rectangular substrates with a width of 25 μm and length from 50 to 100 and 200 μm, respectively. We found that as the cell volume increased, ASMCs exhibited a pro-contractile function with increased mRNA expression of contractile proteins, increased cell stiffness and traction force, and enhanced response to contractile stimulation. We also uncovered a concomitant increase in membrane tension and Piezo1 mRNA expression with increasing cell volume. Perhaps more importantly, we found that the enhanced contractile function due to cell volume increase was largely attenuated when membrane tension and Piezo1 mRNA expression were downregulated, and an auto-regulatory loop between Piezo1 and YAP mRNA expression was also involved in perpetuating the contractile function. These findings, thus, provide convincing evidence of a direct link between hypertrophy and enhanced contractile function of ASMCs that was mediated via Piezo1 mRNA expression, which may be specifically targeted as a novel therapeutic strategy to treat pulmonary diseases associated with ASMC hypertrophy such as severe asthma.

## 1. Introduction

Asthma is a pulmonary disease that affects more than 300 million people worldwide, and 5~10% of the patients develop into severe cases with poor symptom control and heightened risk of exacerbation, hospitalization, and death [1,2,3]. The characteristic features of severe asthma include not only airway hyperresponsiveness (AHR) and inflammation but also airway tissue remodeling, most notably an increased amount of airway smooth muscle [4]. However, the increased amount of airway smooth muscle can arise due to either increased total cell number (hyperplasia) or increased single-cell volume size (hypertrophy) of the airway smooth muscle cells (ASMCs).

Clinical studies have found hyperplasia but not hypertrophy of ASMCs as a pathologic characteristic of patients with mild to moderate asthma [5]. In contrast, patients with severe asthma usually show hypertrophic ASMCs in proximal airways as a selective determinant of disease severity [6]. Clinical studies also showed that patients with severe asthma had more exacerbations during inhaled methacholine to assess airway responsiveness as compared to those with mild to moderate asthmatics [7]. These findings clearly indicate that severe asthma is associated with hypertrophic ASMCs, but whether the hypertrophy leads to hyper-contractile ASMCs as a contributing mechanism for AHR is less clear. Previous studies have used in vitro models to assess the effects of cell size in terms of cell diameter or area on the expression of contractile biomarkers of ASMCs [8,9,10,11,12]. These studies, however, do not sufficiently establish an unambiguous association between the enhanced contractility and the hypertrophy of ASMCs because increased diameter and area do not necessarily represent the increased volume of the cell.

On the other hand, cell volume is determined by a balance between internal and external forces, and, therefore, it affects the cytoskeleton, membrane tension, and mechanosensitive ion transporters/channels located in the membrane [13,14]. Several molecules, such as Desmin, have been found to play important roles in mediating the hypertrophy of ASMCs [12,15,16,17]. Physical stretch and inflammatory chemical actors such as TGF-β and endothelin have also been shown to modulate cell size of in vitro cultured ASMCs [8,9,11,18]. However, there are still no drugs targeting these molecules for clinical trials, largely due to the limitations of these studies in establishing the relationship and underlying mechanism between the cell volume size and contractile function of ASMCs. More recent studies in various cell types have shown that the mechanosensitive ion channel Piezo1 is highly regulated to drive Ca^2+^ signaling by cell volume-dependent membrane tension, suggesting that Piezo1 may be essential in relating the hypertrophy and hyper-contractility of ASMCs, and, thus, a potential target for treating hypertrophy-related AHR in severe asthma [19,20,21,22]. For example, in skeletal muscle, Piezo1 expression decreased with progression of muscle atrophy (decrease in volume) [20,21]. In contrast, Piezo1 expression was upregulated with increasing cell stiffness and contractility in human embryonic kidney cells and pulmonary artery smooth muscle cells [23,24].

Additionally, yes-associated protein (YAP) is also known to be involved in modulating cell volume in combination with cytoskeletal tension [25]. And several studies have indicated that Piezo1 may crosstalk with YAP in the regulation of cell volume and contractile function. For example, Piezo1 and YAP have been shown to play an integrated role in the regulation of vascular smooth muscle contractile function via miR-15b/16 and jagged 2 [26,27]. Piezo1 has also been shown to activate YAP to promote osteogenic differentiation in the aortic valve [28]. Knockdown of Piezo1 appeared to inhibit flow-induced YAP expression in human umbilical vein epithelial cells [29]. On the other hand, it has been shown in oral squamous carcinoma cells that YAP signaling could regulate Piezo1 expression to promote cell proliferation, which seemed to indicate that Piezo1 was a downstream effector molecule of YAP [30,31].

Inspired by these findings, we, thus, hypothesized that in ASMCs, the increase in single-cell volume could result in enhanced cell contractile function through regulation of Piezo1 and YAP crosstalk signaling, which might provide a direct mechanistic link between hypertrophy and hyper-contractility of ASMCs that might ultimately lead to AHR in severe asthma. To test this hypothesis, we used the micropatterning technique to establish an in vitro ASMC hypertrophy model, in which the cell volume size was manipulated by changing the length of the micropattern. Using this model, we showed that with increasing cell volume size, ASMCs became increasingly more contractile, i.e., with increases in expression of contractile biomarkers, cell stiffness, traction force, and response to contractile agonists such as acetylcholine (ACh). We also showed that cytoskeleton organization and membrane tension were required for maintaining the volume-size-regulated contractile function of ASMCs. Furthermore, we found that the hypertrophic ASMCs perpetuated their enhanced contractile function through an auto-regulatory loop of YAP and Piezo1 mRNA expression. Taken together, our results demonstrated that the single-cell volume size was indeed a strong regulator of the contractile function of ASMCs, and the contractile function of hypertrophic ASMCs was perpetuated by Piezo1 in crosstalk with YAP. This provides not only evidence of a direct causative relationship between single-cell volume size and contractility, which was mediated through Piezo1-YAP signaling in ASMCs but also insights for developing potential druggable therapies targeting the ASMC hypertrophy for treating chronic airway diseases such as severe asthma.

## 2. Materials and Methods

### 2.1. Cell Cultures and Reagents Treatment

Primary human ASMCs (#BNCC339826, BeNa, Beijing, China) were cultured in the Dulbecco’s modified Eagle’s medium (DMEM, #11885092, Gibco, Waltham, MA, USA) supplemented with 10% fetal bovine serum (FBS, #16000-044, Gibco, Waltham, MA, USA), 100 units/mL penicillin and streptomycin (#15140122, Gibco, Waltham, MA, USA) in an incubator containing 5% CO_2_ humidified at 37 °C and used at passages 3–8 with normal morphology and contractile functions. Cells were verified by immunostaining using the antibody against α-SMA (a marker of ASMCs) and showed a typical “hill and valley” appearance under phase-contrast microscopy.

For reagents treatment, cells were respectively treated with 1 μM Blebbistatin (Ble, #HY-13813, MCE, Shanghai, China), 1 μM Y27632 (#HY-10071, MCE, Shanghai, China), and 10 μM ML-7 (#HY-15417, MCE, Shanghai, China) for 1 h. For contractile stimulation, 100 μM acetylcholine (ACh, #HY-B0282, MCE, Shanghai, China) was used.

### 2.2. Micropatterning

Briefly, polydimethylsiloxane (PDMS, Dow Corning, Midland, MI, USA) prepolymer was mixed in a 1:10 ratio of curative-to-precursor according to the manufacturer’s protocol and added to replica molding against a silicon mold made by photolithography [32]. An amount of 50 μg/mL fibronectin (FN, #33016015, Gibco, Waltham, MA, USA) was allowed to adsorb onto the surface of each PDMS stamp. Then, the PDMS stamp was deposited onto the surface of the substrate to allow for a complete protein transfer. Next, 1% Pluronic F127 (#P2443, Sigma, St. Louis, MO, USA) solution was added to the substrates for 1 h to passivate non-FN-coated regions.

### 2.3. Immunofluorescence

For immunofluorescent staining, ASMCs were rinsed twice with phosphate-buffered saline (PBS), followed by fixation using 3.7% paraformaldehyde (#C104190, Aladdin, Shanghai, China) for 10 min. Then, cells were washed and permeabilized with 0.5% Triton X-100 (#P0096, Beyotime, Shanghai, China) for 15 min. After washing twice with PBS, the cells were treated with 10% bovine serum albumin (BSA, blocking solution) for 1 h. This was followed by incubation with required primary antibodies (in blocking buffer). The primary antibodies were the following: Piezo1 (1:100; #PA5-72974, Invitrogen, Waltham, MA, USA); Caveolin 2 (1:100; Invitrogen, MA5-32083, Waltham, MA, USA); YAP (1:100; #SAB5700948, Sigma, St. Louis, MO, USA); and α-SMA (1:100; #BM0002, Boster, Wuhan, China). The secondary antibodies were the following: goat anti-rabbit IgG H&L (Alexa Fluor 488) (1:500; #A11008, Invitrogen, Waltham, MA, USA); goat anti-rabbit IgG H&L (Alexa Fluor 546) (1:500; #A11035, Invitrogen, Waltham, MA, USA); and goat anti-mouse IgG H&L (Alexa Fluor 488) (1:500; #A21121, Invitrogen, Waltham, MA, USA). F-actin (red) was stained with fluorescent red phalloidin (1:200, #51927, Sigma, St. Louis, MO, USA) for 20 min, and nuclei (blue) were stained with DAPI (#C1005, Beyotime, Shanghai, China) for 3 min in the dark. Stained cells were visualized by using laser scanning confocal microscopy at 63× objective (LSM710, Zeiss, Oberkochen, Germany). Acquisition parameters were kept constant during the experiments.

### 2.4. Cell Volume Size Measurement

For the ASMCs cultured on the petri dish, the cells were visualized using the Zeiss microscopy system (Zeiss Cell Observer) at 20× objective. The cell width, length, and area of ASMCs were measured by using Image J 1.45.

For the ASMCs cultured on the micropatterns, the cell with F-actin and DAPI staining was recorded at 0.2 μm z-axis intervals by using laser scanning confocal microscopy at 63× objective. A stack of gray-level images (8 bits) was carried out by using ImageJ 1.45 (3D Viewer) to obtain the 3D visualization. The volume of the cell and nucleus was calculated by counting the voxel number after thresholding the stack.

### 2.5. Cell Stiffness Assessment

In brief, ASMCs cultured on the micropatterns were serum-deprived for 12 h and incubated with Arg-Gly-Asp-coated ferrimagnetic microbeads (4.5 μm diameter) for 30 min. Then, unbound beads were washed away with DMEM, magnetized horizontally with a brief 1000-Gauss pulse, and twisted in a vertically aligned homogenous magnetic field (20 Gauss) that was varying sinusoidally in time.

In order to assess the cell stiffness versus cell volume, the cells were twisted in the oscillatory magnetic field at a frequency consecutively increasing from 0.1 to 1000 Hz. In order to evaluate the variation in cell stiffness in response to 100 μM ACh, the cells were performed at a single frequency of 0.75 Hz and first measured for 60 s to achieve baseline stiffness. The dosage of 100 μM ACh was determined and used in all subsequent experiments according to a prior test that estimated the dose–effect relation between ACh and cell stiffness in the cultured ASMCs. Then, ACh was added to the cells, after which the cells were continuously measured for stiffness for up to 330 s. And the effect of the drug on the cell stiffness was quantified by the ratio of the averaged plateau value of cell stiffness in the presence of the ACh treatment to baseline stiffness in the absence of the ACh treatment.

### 2.6. Cell Traction Force Assessment

The substrate for the cell culture for the assessment of traction force was the polyacrylamide gel embedded with fluorescent microbeads (0.2 μm, #F8810, Molecular probes, Eugene, OR, USA). The cells were seeded onto the substrate with different patterns and serum-deprived for 12 h. A single cell and the fluorescent microbeads were then imaged by phase contrast and fluorescence microscopy, respectively. Then, 1 M NaOH was added to cell cultures. After 5 min, the cell-free bead positions were recorded as a reference point (traction-free) for bead displacement. The cell traction force was calculated by converting the displacement of microbeads (i.e., substrate deformation) to the force exerted by the cell on the substrate with different patterns using proprietary MATLAB software (MathWorks Corp., Natick, MA, USA) according to the images of the cells (phase contrast) and the microbeads (fluorescence) obtained before and after the NaOH treatment.

### 2.7. Calcium Signal Imaging

Briefly, ASMCs that cultured on the micropatterns were serum-deprived for 12 h and incubated with 5 μM Fluo-4 acetoxymethylester (Fluo-4/AM, #F14201, Sigma, St. Louis, MO, USA) for 30 min at 37 °C. The cells were then incubated with Tyrode’s solution for 20 min. The fluorescence intensity of the single cell cultured on the micropattern was recorded by laser scanning confocal microscopy (LSM710, Carl Zeiss, Oberkochen, Germany) using an excitation wavelength of 488 nm and an emission wavelength of 505 nm. The cells were first measured for 20 s to achieve baseline fluorescence (F_0_) over certain regions of the cytoplasm. Then, ACh was added to the cells, and the cells were continuously measured for 193 s (F). Subsequent image processing and analysis were performed using Image J 1.45 software. [Ca^2+^]_i_ was represented as F/F_0_.

### 2.8. Plasmid and siRNA Transfections

Briefly, cells (70 to 80% confluent) in six-well plates were transfected with either 50 nM biosensor DNA or small interfering RNA (siRNA, RiboBio, Hangzhou, China) and corresponding scramble controls by using Lipofectamine3000 according to the manual protocol (#2563912, Invitrogen, Waltham, MA, USA). After 8 h incubation, the medium was changed, and cell imaging experiments were performed 48 h later.

### 2.9. Reverse Transcription and qPCR Analyses

Total RNA from cultured ASMCs on the micropattern was extracted using the TRI Reagent RNA Isolation Reagent (#T9424, Sigma-Aldrich, St. Louis, MO, USA). An amount of 500 ng total RNA was used to generate the 1st strand cDNA using the Revert Aid First Strand cDNA Synthesis Kit (#K1622, Thermo, Waltham, MA, USA). Primers (Table S1) were purchased from General Biosystems (Chuzhou, China). PowerUp SYBR Green Master Mix (#A25742, Applied Biosystems, Foster City, CA, USA) was used. The reaction was run in the qRT-PCR system (StepOnePlus, Applied Biosystems, Foster City, CA, USA) using 1 µL of the cDNA in a 10 µL reaction according to the manufacturer’s instructions in triplicate. Calibration and normalization were performed using the 2^−∆∆CT^ method, where ∆∆C_T_ = C_T_ (target gene) − C_T_ (reference gene). Fold changes in mRNA expression were calculated from the resulting C_T_ values from three independent experiments.

### 2.10. FRET Microscopy Imaging and Quantification

Briefly, cells were excited by using a live cell image system (Cell Observer 1A, Carl Zeiss). The parameters for the excitation filter and dichroic mirror were 436 ± 10 and 455 nm, respectively, and the emission filters of ECFP and YPet channels were 480 ± 20 nm and 535 ± 15 nm, respectively. FRET quantifications by calculating YPet/ECFP were processed using the Wang Lab (UCSD)-developed software package FluoCell in MATLAB (available at http://github.com/lu6007/fluocell accessed on 14 October 2023). Fluorescence signals from ECFP and YPet images were measured after background subtractions, and the ratio of the two channels was calibrated in a pixel-to-pixel manner.

### 2.11. Atomic Force Microscope Study

Cell membrane stiffness (Young’s modulus) was characterized at the nanometer scale using a Nanowizard II atomic force microscope (AFM, JPK Instruments AG, Berlin, Germany) mounted on an Olympus IX 81 inverted light microscope, as described previously [33]. The tip of the cantilever (0.03 N/m) was pushed into the cell surface, and the indentation depth and the deflection of the cantilever were recorded at an approach speed of 2 μm/s and a maximum set point of 0.1 V. Then, Young’s modulus was obtained by fitting the force–indentation curve to the Hertz model, where force was determined by multiplying the deflection by the spring constant of the cantilever following Hooke’s law.

### 2.12. Statistical Analysis

The data are reported as means ± SEM. Comparisons between multiple groups were analyzed using one-way ANOVA with a Tukey post hoc test, and two-group comparisons were analyzed using the unpaired Student’s *t*-test. The statistical analyses were performed using GraphPad Prism 9.0. Statistically significant differences are indicated as follows: * for *p* < 0.05 and ** for *p* < 0.01.

## 3. Results

### 3.1. Increase in Cell Volume Promotes Contractile Function of ASMCs

The volume size of individual ASMCs was manipulated by using micropatterning technology as adapted from the published literature [34]. We first accessed the size distribution of ASMCs cultured in low density on the petri dish by using optical microscopy. ASMCs grown in this condition could spread freely on the substrate, resulting in variable cell width, length, and area. As shown in Figure S1 of 330 ASMCs measured, their width was distributed narrowly from 20 to 30 μm, and their length spanned from 40 to 240 μm. Therefore, the cell area averaged at about 1000~2000 μm^2^ but could reach 5000~6000 μm^2^ for very large cells. These results indicated that ASMCs maintained almost constant width but largely increased in length while growing in size.

**Figure 1:**
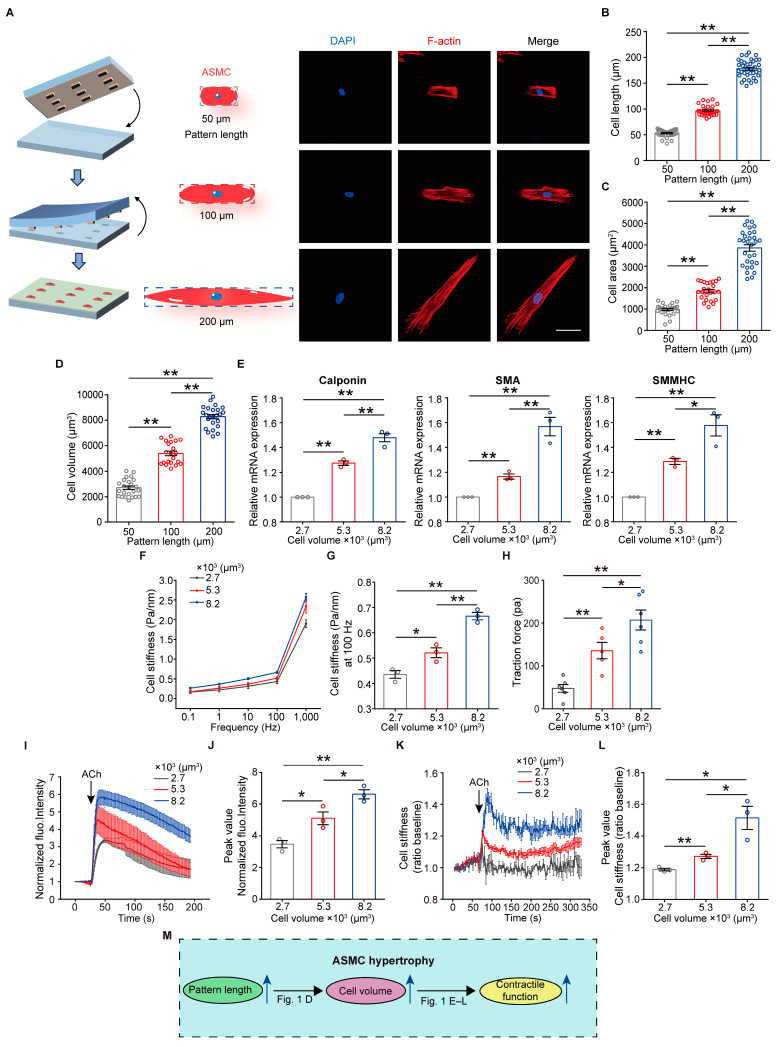
Effects of cell volume manipulated by micropatterning technology on change in the contractile function of ASMCs. (**A**) Schematic illustration of micropatterning for cell volume manipulation and representative images of fluorescently stained (red: F-actin, blue: DAPI for nucleus) single ASMC grown on the micropattern of same width (25 μm) but different lengths (50, 100, and 200 μm). (**B**–**D**) Quantified cell length, area, and volume of ASMCs grown on the micropatterns with lengths of 50, 100, and 200 μm, respectively (n = 20–30 cells). (**E**) Quantitative RT-PCR measured mRNA expression of contractile proteins (calponin, SMA, and SMMHC) versus cell volume of ASMCs (n = 3). (**F**) Cell stiffness of ASMCs measured by OMTC at 0.1, 1.0, 10.0, 100.0, and 1000.0 Hz versus cell volume (n = 3). (**G**) Quantification of cell stiffness of ASMCs measured by OMTC at 100 Hz versus cell volume (n = 3), as shown in (**F**). (**H**) Cell traction force of ASMCs measured by FTTFM versus cell volume (n = 5–6 cells). (**I**) Time courses of changing normalized fluorescence intensity of Fluo-4/AM of cultured ASMCs in response to 100 µM ACh (n = 10–12 cells from 3 experiments). (**J**) Quantification of the peak values of the released calcium, as shown in (**I**). (**K**) Time courses of changing normalized cell stiffness of cultured ASMCs in response to 100 µM ACh (n = 3). (**L**) Quantification of the peak values of the cell stiffness, as shown in (**K**). (**M**) Schematic diagram of correlation between micropattern length, cell volume, and contractile function of cultured ASMCs. Data are means ± S.E.M. Scale bar = 50 μm. * *p* < 0.05; ** *p* < 0.01.

Therefore, we followed this phenomenon to design our in vitro micropatterns to grow ASMCs into different sizes by only changing the pattern length in vitro. In accordance with the above cell size distribution, the micropatterns were designed as rectangles with widths always being 25 μm but lengths being either 50, 100, or 200 μm, and fabricated on elastic PDMS substrates with fibronectin coating (Figure S2). When cultured on the micropatterns, the ASMCs grew within only the restricted rectangular areas with different engineered lengths, which were confirmed by fluorescent microscopy of the ASMCs with F-actin staining (Figure 1A). The length, area, and volume size of the ASMCs grown on the micropatterns were quantified using stack imaging laser scanning confocal microscopy and ImageJ analysis. As shown in Figure 1B, ASMCs, by and large, grew to the full length of the engineered micropatterns. Cells grown on the micropatterns of 200 μm length could reach a maximal area of around 5000 μm^2^, close to the largest ASMCs grown on the petri dish (Figure 1C). Most importantly, as the pattern length increased from 50 to 100 and 200 μm, the average cell volume of the ASMCs increased from 2700 to 5300 and 8200 μm^3^, respectively, indicating a maximal 3.0/1.4 times hypertrophy for ASMCs on 200 μm long micropatterns compared to their counterparts on 50/100 μm long micropatterns (Figure 1D).

To investigate the effect of cell volume on the expression of contractile proteins, we analyzed the expression of smooth muscle contractile markers, such as calponin, smooth muscle α-actin (SMA), and smooth muscle myosin heavy chain (SMMHC), using quantitative PCR. The results showed that the expression of calponin, SMA, and SMMHC all increased with increasing cell volume (Figure 1E). Quantification of the fluorescent intensity of ASMCs with SMA and phosphorylated myosin light chain 2 (P-MLC) fluorescence staining also revealed the same trend of increasing SMA and P-MLC expression versus cell volume (Figure S3).

To further analyze the effect of cell volume on the contractile function of ASMCs, we performed optical magnetic twisting cytometry (OMTC) and Fourier transformation traction force microscopy (FTTFM) to assess cell stiffness and traction force of ASMCs in different volume sizes. We found that the cell stiffness of the ASMCs appeared to increase with increasing cell volume (Figure 1F). As shown quantitatively in Figure 1G, the cell stiffness of ASMCs increased from 0.44 to 0.52 and 0.67 Pa/nm as the cell volume increased from 2.7 to 5.3 and 8.2 × 10^3^ μm^3^ (*p* < 0.01). Similarly, the traction force of the ASMCs increased markedly with increasing cell volume (Figure 1H).

The effect of cell volume on the contractile function of ASMCs was further explored in terms of intracellular calcium influx ([Ca^2+^]_i_) induced by ACh (a contractile parasympathetic neurotransmitter in the airway). [Ca^2+^]_i_ was evaluated by changes in fluorescent intensity of the micropatterned ASMCs with Fluo-4/AM staining before and after exposure to 100 μM ACh. As shown in Figure 1I,J and Figure S4A, the response of ACh-induced [Ca^2+^]_i_ in ASMCs increased as the cell volume increased (2.7 vs. 5.2, 8.2 × 10^3^ μm^3^). Moreover, we found that the baseline calcium fluorescence decreased with increasing cell volume (Figure S5). In addition, ASMCs responded to ACh with stiffness spikes, which appeared to be enhanced as the cell volume increased (Figure 1K,L and Figure S4B). These results indicated that larger ASMCs were more responsive to contractile agonist stimulation. Taken together, the above findings demonstrated that as cell volume was upregulated by pattern length, ASMC exhibited a pro-contractile function with an increase in contractile proteins, cell stiffness, traction force, and contractile responsiveness in association with ASMC hypertrophy (Figure 1M).

### 3.2. Cell Volume Regulates Perinuclear Cytoskeleton Organization and Membrane Tension of ASMCs

**Figure 2:**
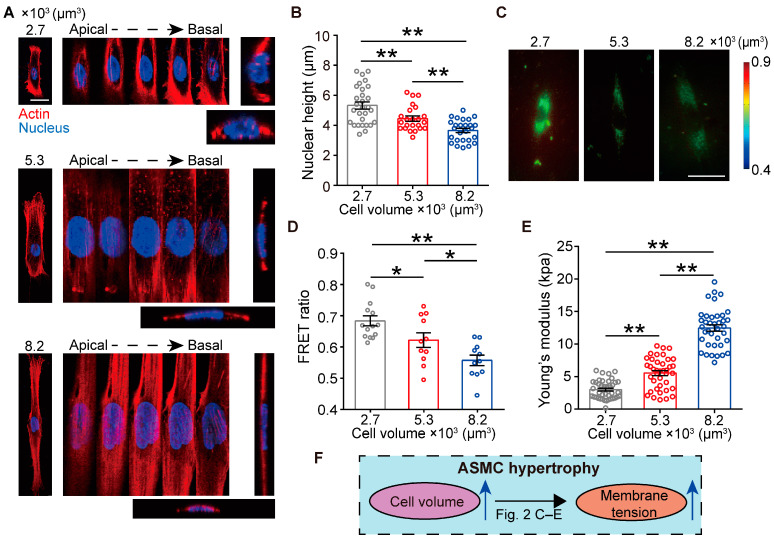
Effects of cell volume on the cytoskeleton, nuclear size, and membrane tension. (**A**) Representative images of the actin structure of single ASMC with cell volume of 2700, 5300, or 8200 μm^3^, respectively. Each cell was imaged at different planes from the apical to basal plane together with orthogonal size views. (**B**) Quantification of the height of the ASMCs nucleus versus the cell volume (n = 20–28 cells). (**C**) Representative FRET ratio images of ASMCs at different cell volumes and the fluorescence intensity reflected membrane tension. (**D**) Quantified FRET ratio in ASMCs versus cell volume (n = 11–14 cells). (**E**) Cell membrane stiffness measured as Young’s Modulus by AFM versus cell volume (n = 38–40 from 10–12 cells). (**F**) Schematic diagram of the changing cell volume and membrane tension in ASMC hypertrophy. Data are means ± S.E.M. Scale bar = 50 μm. * *p* < 0.05; ** *p* < 0.01.

We then assessed the effects of cell volume on cytoskeleton organization, particularly the 3D structure of actin stress fibers and the nuclear morphology of ASMCs. As shown in Figure 2A, the increase in cell volume resulted in the remodeling of the perinuclear actin organization from a mesh-like structure to distinct parallel fibers along the long axis and above the nucleus of the cell. In some of the largest cells (8200 μm^3^), the actin fibers appeared to be broken down around the nucleus. These results of cytoskeleton reorganization were in line with our previous findings of the contractile functions, such as cell stiffness and traction force dependence of cell volume. In addition, as the cell volume increased, the nucleus became increasingly elongated along the cell’s long axis and more flattened, as shown in the orthogonal cross-sectional images of the cells, which indicated that there existed a force that derived from the cytoskeleton to compact the nucleus as the volume increased. Quantitative imaging analysis found that the nuclear height decreased from ~5.3 to ~4.4 and ~3.6 μm, respectively, while the nuclear area consistently increased when the cell volume increased from 2700 to 5300 and 8200 μm^3^ (Figure 2B and Figure S6A). Consequently, the nuclear volume did not seem to change with cell volume (Figure S6B).

On the other hand, it has been shown before that the increase in cell volume during osmosis is coupled to cell membrane tension, which reflects the forces contributed by cytoskeleton reorganization [35]. To investigate whether cell volume would influence membrane tension in our case, we detected membrane tension of the cultured ASMCs using a fluorescence resonance energy transfer (FRET)-based membrane tension biosensor [36]. As shown in Figure 2C,D, we observed a significant decrease in the FRET ratio of ASMCs as the cell volume increased from 2700 to 8200 μm^3^, indicating increasing membrane tension during cell size growth on the micropatterns. To confirm these results, we used AFM to probe the elasticity of the membrane [37]. Cell membrane stiffness increased from ~2.99 kPa to ~5.54 kPa and ~12.46 kPa when the cell volume increased from 2700 to 5300 and 8200 μm^3^ (Figure 2E), consistent with our previous results. These results suggested that cell volume increase (hypertrophy) could lead to altered cytoskeleton structure and membrane tension of ASMCs (Figure 2F).

### 3.3. Membrane Tension Is Required for Cell Volume to Regulate Contractile Function of ASMCs

**Figure 3:**
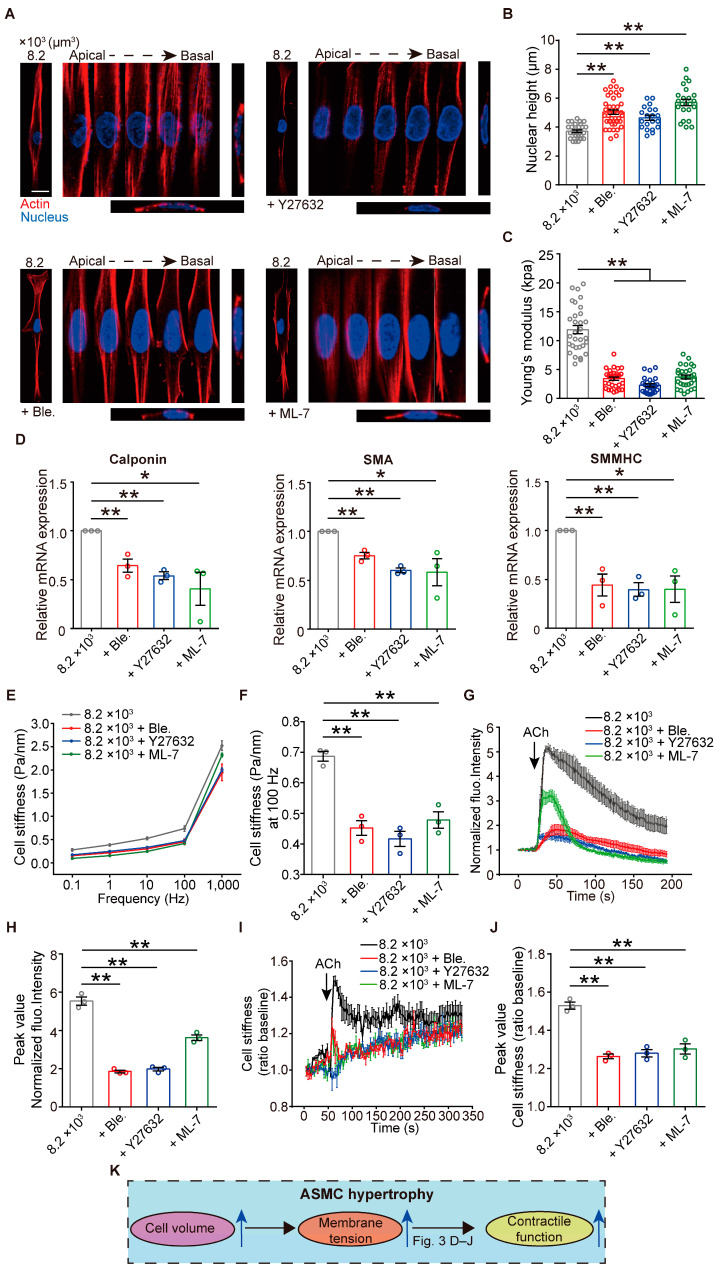
Effects of membrane tension on the cell volume-regulated contractile function of ASMCs. (**A**) Representative images of the actin structure of single ASMCs with cell volume of 8200 μm^3^ before and after treatment with 1 μM Ble, 1 μM Y27632, and 10 μM ML-7, respectively. Each cell was imaged at different planes from the apical to basal plane, together with orthogonal side views. (**B**–**D**) Quantification of nuclear height (n = 20–30 cells), cell membrane stiffness (n = 30 from 10–12 cells), and qPCR-detected mRNA expression of contractile proteins (calponin, SMA, and SM-MHC, n = 3) of the cells with or without pretreatment of Ble, Y27632, and ML-7, respectively. (**E**) Cell stiffness measured by OMTC at 0.1, 1.0, 10.0, 100.0, and 1000.0 Hz with or without pretreatment of Ble, Y27632, and ML-7, respectively (n = 3). (**F**) Quantification of cell stiffness of ASMCs measured by OMTC at 100 Hz versus cell volume (n = 3), as shown in (**E**). (**G**) Time courses of changing normalized fluorescence intensity of Fluo-4/AM of cultured ASMCs (8200 μm^3^) in response to 100 µM ACh with or without pretreatment of Ble, Y27632, and ML-7, respectively (n = 10–12 cells from 3 experiments). (**H**) Quantification of the peak values of the released calcium, as shown in (**G**). (**I**) Time courses of changing cell stiffness of cultured ASMCs (8200 μm^3^) in response to 100 µM ACh with or without pretreatment of Ble, Y27632, and ML-7, respectively (n = 3). (**J**) Quantification of the peak values of the cell stiffness, as shown in (**I**). (**K**) Schematic diagram of the changing cell volume and contractile function promotion via membrane tension during ASMC hypertrophy. Data are means ± S.E.M. Scale bar = 50 μm. * *p* < 0.05; ** *p* < 0.01.

Membrane tension has been shown to regulate many crucial cell functions, including morphogenesis, motility, endocytosis, and mechanosensation [14]. Its role in promoting contractile function due to ASMC hypertrophy is, however, underappreciated. Therefore, we evaluated the involvement of membrane tension in contractile function regulation by cell volume increase. To decrease the membrane tension of the hypertrophic ASMCs with a cell volume of 8200 μm^3^, we treated the cell with either 1 μM Ble (a myosin II ATPase inhibitor), 1 μM Y-27632 (a Rho kinase inhibitor), or 10 μM ML-7 (a myosin II light chain kinase inhibitor) [38]. The results showed that compared to non-treated cells (8.2 × 10^3^), ASMCs treated with Ble, Y-27632, and ML-7 exhibited significantly disrupted actin organization, increased nuclear height, and decreased cell membrane stiffness, suggesting that these three inhibitors significantly decreased membrane tension of the hypertrophic ASMCs (Figure 3A–C).

Then, we further found that the mRNA expression of contractile protein (calponin, SMA, and SMMHC) in ASMCs with cell volume of 8200 μm^3^ was also significantly reduced after the treatment with Ble, Y-27632, or ML-7 (Figure 3D). Cell stiffness, ACh-induced Ca^2+^ release, and ACh-induced cell stiffness increase in ASMCs with cell volume of 8200 μm^3^ were all attenuated to various extents after the treatment with Ble, Y-27632, or ML-7 (Figure 3E–J and Figure S4C,D). These results together implicated that membrane tension plays a significant role in the upregulation of contractile function by cell volume increase (Figure 3K).

### 3.4. Piezo1 Mediates the Cell Volume-Regulated Contractile Function of ASMCs

**Figure 4:**
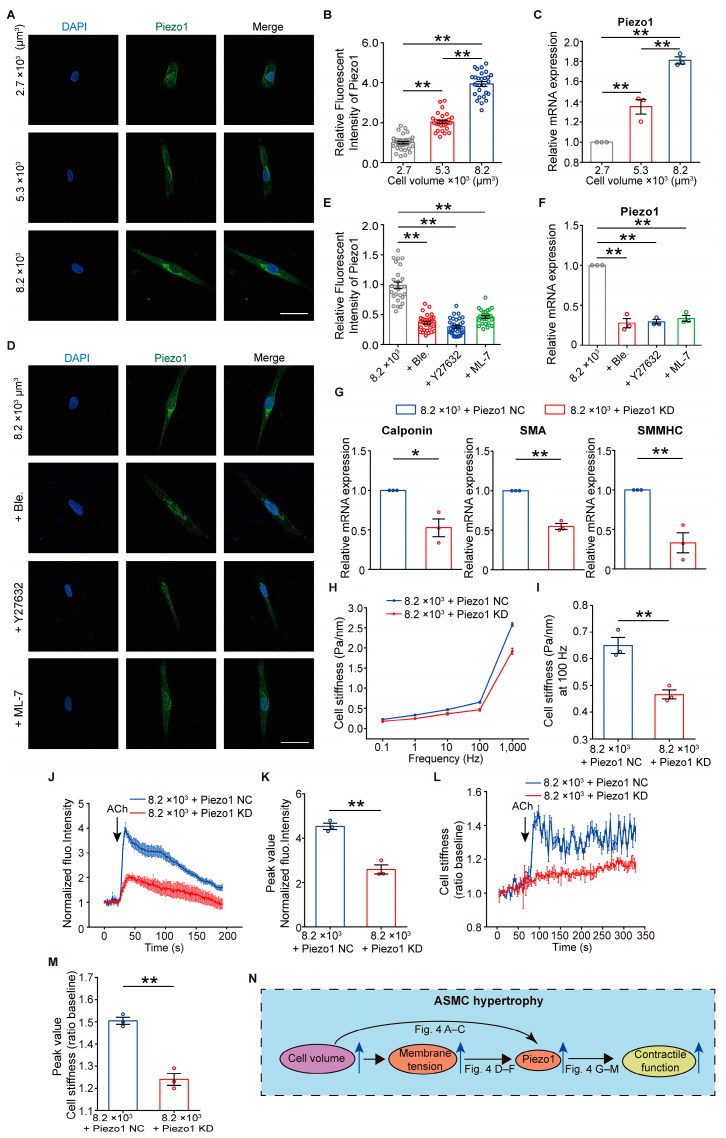
Effects of Piezo1 on the contractile function of hypertrophic ASMCs. (**A**) Representative images of ASMCs with immunofluorescence staining of Piezo1 at different cell volumes (2.7, 5.3, and 8.2 × 10^3^ μm^3^, respectively). (**B**) Quantified Piezo1 fluorescent intensity of ASMCs versus cell volume (n = 27–30 cells). (**C**) The mRNA expression of Piezo1 in ASMCs versus cell volume (n = 3). (**D**) Representative images of immunofluorescent staining of Piezo1 in the hypertrophic ASMCs (8.2 × 10^3^ μm^3^) either not or pretreated with 1 μM Ble, 1 μM Y27632, and 10 μM ML-7. (**E**) Quantified Piezo1 fluorescent intensity of hypertrophic ASMCs (8.2 × 10^3^ μm^3^) either not or pretreated with 1 μM Ble, 1 μM Y27632, and 10 μM ML-7 (n = 27–30 cells). (**F**) The mRNA expression of Piezo1 in the hypertrophic ASMCs (8.2 × 10^3^ μm^3^) either not or pretreated with Ble, Y27632, and ML-7 (n = 3). (**G**) The mRNA expression of contractile proteins (calponin, SMA, and SM-MHC) in the hypertrophic ASMCs (8.2 × 10^3^ μm^3^) pretreated with either Piezo1 negative control (NC) or knockdown (KD) siRNA (n = 3). (**H**) Cell stiffness of the hypertrophic ASMCs (8.2 × 10^3^ μm^3^) pretreated with either Piezo1 NC or KD siRNA, which was measured by OMTC at 0.1, 1.0, 10.0, 100.0, and 1000.0 Hz (n = 3). (**I**) Quantification of cell stiffness of ASMCs measured by OMTC at 100 Hz versus cell volume (n = 3), as shown in (**H**). (**J**) Time courses of changing normalized fluorescence intensity of Fluo-4/AM in the hypertrophic ASMCs (8.2 × 10^3^ μm^3^) pretreated with either Piezo1 NC or KD siRNA in response to 100 µM ACh (n = 10–12 cells from 3 experiments). (**K**) Quantification of the peak values of the released calcium, as shown in (**J**). (**L**) Time courses of changing cell stiffness of cultured ASMCs (8200 μm^3^) pretreated with either Piezo1 NC or KD siRNA in response to 100 µM ACh (n = 3). (**M**) Quantification of the peak values of the cell stiffness, as shown in (**L**). (**N**) Schematic diagram of the role of Piezo1 in regulation of contractile function during ASMC hypertrophy. Data are means ± S.E.M. Scale bar = 50 μm. * *p* < 0.05; ** *p* < 0.01.

In view of the important role of mechanosensitive ion channel Piezo1 in sensing membrane tension and regulating cell functions [39,40], we further explored whether the cell volume-enhanced contractile function was associated with the upregulation of Piezo1 expression and, thereby, could be ameliorated by pharmacologically targeting Piezo1 of hypertrophic ASMCs. For this purpose, we first evaluated Piezo1 expression in ASMCs of different cell volumes by immunofluorescent microscopy. We found that Piezo1 was enriched in the perinuclear area as the cell volume increased (Figure 4A). Quantification of the Piezo1 fluorescent intensity of ASMCs revealed the same trend of increasing Piezo1 expression versus cell volume (Figure 4B). This trend was probably related to the caveolae-dependent endocytosis (Figure S7). In addition, quantitative PCR results also demonstrated that mRNA expression of Piezo1 increased significantly as the cell volume increased from 2700 to 8200 μm^3^ (Figure 4C). The enhanced Piezo1 expression in the hypertrophic ASMCs (cell volume: 8200 μm^3^) was largely abolished by pretreating the cells with Ble, Y27632, and ML-7 to reduce the membrane tension, respectively (Figure 4D–F).

Furthermore, we demonstrated that Piezo1 mRNA expression in the hypertrophic ASMCs was knocked down with small interfering RNA (siRNA) (Figure S8A), which reduced the mRNA expression of contractile markers (calponin, SMA, and SMMHC) by more than 50% (8200 + Piezo1 NC vs. 8200 + Piezo1 KD) (Figure 4G). Piezo1 knockdown also significantly reduced the cell stiffness and attenuated the ACh-induced Ca^2+^ release as well as the ACh-induced contraction (cell stiffness ratio to baseline) in the hypertrophic ASMCs (Figure 4H–M and Figure S4E,F). These results, thus, highlighted a key role of Piezo1 mRNA expression in regulating the cell volume-dependent contractile function in ASMC hypertrophy (Figure 4N).

### 3.5. Piezo1 Mediates the Cell Volume-Regulated Contractile Function in ASMCs via a YAP Auto-Regulatory Loop

**Figure 5:**
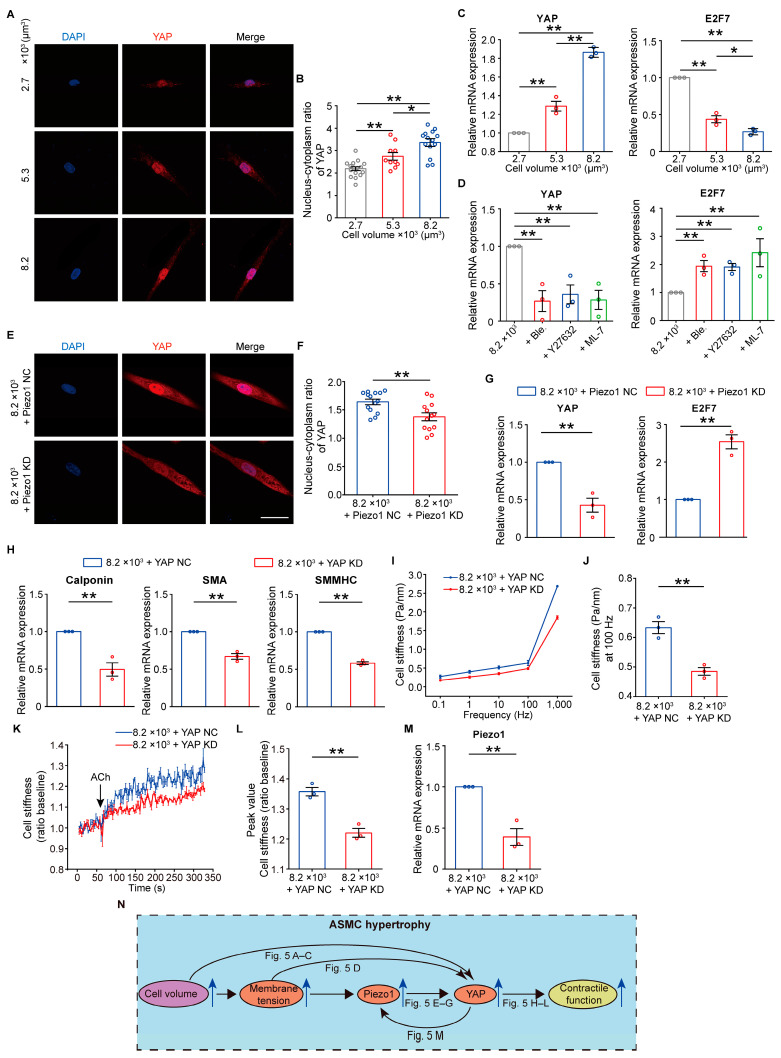
Effects of YAP on the contractile function of hypertrophic ASMCs. (**A**,**B**) Representative images and quantified nucleus–cytoplasm fluorescence intensity ratio of ASMCs with immunofluorescence staining of YAP at different cell volumes (2.7, 5.3, or 8.2 × 10^3^ μm^3^; n = 15–20 cells). (**C**) The mRNA expression of YAP and E2F7 in ASMCs versus cell volume (2.7, 5.3, or 8.2 × 10^3^ μm^3^; n = 3). (**D**) The mRNA expression of YAP and E2F7 in the hypertrophic ASMCs (8.2 × 10^3^ μm^3^) either not or pretreated with Ble, Y27632, and ML-7 (n = 3). (**E**–**G**) Representative images, quantified nucleus-cytoplasm intensity ratio of immunofluorescent YAP staining, and the mRNA expression of YAP and E2F7 in the hypertrophic ASMCs (8.2 × 10^3^ μm^3^) transfected with either Piezo1 NC or Piezo1 KD siRNA (n = 13–15 cells). (**H**) The mRNA expression of contractile proteins (calponin, SMA, and SM-MHC) in the hypertrophic ASMCs (8.2 × 10^3^ μm^3^) pretreated with either YAP NC or KD siRNA (n = 3). (**I**) Cell stiffness of the hypertrophic ASMCs (8.2 × 10^3^ μm^3^) pretreated with either YAP NC or KD siRNA, which was measured by OMTC at 0.1, 1.0, 10.0, 100.0, and 1000.0 Hz (n = 3). (**J**) Quantification of cell stiffness of ASMCs measured by OMTC at 100 Hz versus cell volume (n = 3), as shown in (**I**). (**K**) Time courses of changing cell stiffness of cultured ASMCs (8200 μm^3^) pretreated with either YAP NC or KD siRNA in response to 100 µM ACh (n = 10–12 cells from 3 experiments). (**L**) Quantification of the peak values of the cell stiffness, as shown in (**K**). (**M**) The mRNA expression of Piezo1 in the hypertrophic ASMCs (8.2 × 10^3^ μm^3^) transfected with either YAP NC or YAP KD siRNA (n = 3 in all cases). (**N**) Schematic diagram of the involvement of cell volume, Piezo1, and YAP in the regulation of contractile function of ASMCs during hypertrophy. Data are means ± S.E.M. Scale bar = 50 μm. * *p* < 0.05; ** *p* < 0.01.

Piezo1 has also been shown to regulate the nuclear localization of YAP, which suggests that YAP activated by Piezo1 may be involved in the regulation of contractile function of ASMCs during hypertrophy [41,42]. Indeed, the immunofluorescent microscopy assay showed the increasing perinuclear localization of YAP in ASMCs with the increasing cell volume (typical images and quantified nucleus-cytoplasm ratio of YAP level in Figure 5A,B). qPCR assay showed a similar fashion of the increasing mRNA expression of YAP in ASMCs with the increasing cell volume (Figure 5C left panel), whereas the mRNA expression of transcription factor E2F7 decreased with the increasing cell volume (Figure 5C right panel) in agreement with another report [43].

Modulation of membrane tension by pretreatment with Ble, Y-27632, or ML-7 completely reversed the cell volume-induced response of YAP and E2F7 mRNA expression in ASMCs (Figure 5D). Knockdown of Piezo1 expression with siRNA in hypertrophic ASMCs (8.2 × 10^3^ μm^3^) seemed to significantly arrest the perinuclear translocation of YAP (8.2 × 10^3^ + Piezo1 NC vs. 8.2 × 10^3^ + Piezo1 KD, typical images and quantified nucleus-cytoplasm ratio of YAP level in Figure 5E,F), together with reversion of YAP and E2F7 mRNA expression in the hypertrophic ASMCs (Figure 5G).

To further confirm the functional relevance of YAP with the contractile function of ASMC hypertrophy, we transfected the hypertrophic ASMCs (8.2 × 10^3^ μm^3^) with siRNA to inhibit YAP expression (Figure S8B). Consequently, the mRNA expression of calponin, SMA, and SMMHC was markedly lowered (8.2 × 10^3^ + YAP NC vs. 8.2 × 10^3^ + YAP KD, Figure 5H). Similarly, YAP knockdown led to decreased cell stiffness, reduced stiffness response to ACh stimulation, and Piezo1 mRNA expression in the hypertrophic ASMCs (Figure 55I–M and Figure S4G). These results suggested that there might be an auto-regulatory loop of YAP through which Piezo1 regulated contractile function as cell volume increased (Figure 5N).

## 4. Discussion

In the present study, we used the micropatterning technique to manipulate single-cell volume by changing the length of rectangular micropatterns to mimic ASMC hypertrophy in vitro. The pattern length was selected in accordance with the length distribution of ASMCs cultured in the petri dish without constraint, which could characterize the cells in small, average, and large sizes with lengths of 50, 100, and 200 μm, respectively. The pattern width was designed as 25 μm in all cases to ensure only a single cell grown to average width on each single micropattern. When cultured on these micropatterns with widths of 25 μM and lengths of 50, 100, and 200 μm, the single-cell volume, on average, was 2700, 5200, and 8200 μm^3^, indicating a maximal 3.0-fold increase in cell volume between the cells grown on 200 μm long micropatterns and those on 50 μm long micropatterns, which was consistent with the cell volume in ASMC hypertrophy described in previous reports [6,44]. These single cells were in a non-proliferative but contractile phenotype due to both the micropattern constraint and serum deprivation prior to the experiment.

Cell volume is thought to be a well-controlled cellular characteristic that affects multiple cellular functions, including cell mechanics [45]. Some studies hold the view that there is a negative relationship between cell volume and contraction. For example, Guo et al. reported that cell stiffness increased concomitantly with cell volume decrease [34]. Liu et al. further showed that cell traction force increased/decreased as the cell shrank/swelled [46]. In these studies, the cell volume was regulated by changing osmotic pressure to cause water influx/efflux and, thus, inflate/deflate the cells, which was a rapid process occurring on small time scales, typically within seconds [34]. The pathological process of ASMC hypertrophy is, however, much slower and usually develops on time scales of hours, if not even longer, as the cell needs that time to grow its plasma membrane, DNA, proteins, and other intracellular materials [47]. Therefore, we regulated the cell volume of homologous ASMCs by culturing them on micropatterns of different lengths, which successfully established a suitable model for studying ASMC hypertrophy in vitro.

With this model, we found that the hypertrophic ASMCs with single-cell volume of 8200 μm^3^ exhibited a pro-contractile function with increased mRNA expression of key contractile proteins, increased cell stiffness and traction force, and enhanced stiffness response to contractile agonists such as ACh. We also observed concomitant cytoskeleton reorganization and membrane tension alteration, which were required for contractile function regulation of ASMCs due to cell volume increase. In addition, we discovered that cell volume regulated the mRNA expression of Piezo1, which is sensitive to membrane tension, and an auto-regulatory loop between Piezo1 and YAP was involved in the regulation of contractile function during ASMC hypertrophy. These findings uncovered a novel mechanism of contractile function enhancement solely due to cell volume increase in ASMC hypertrophy, which involved crosstalk between Piezo1 and YAP and, thus, might provide a potential therapeutic strategy for treating pulmonary diseases associated with ASMC hypertrophy by specifically targeting the cell’s Piezo1 channel.

In this study, we focused on the role of membrane tension in the cell volume-enhanced contractile function because cell volume is known to be regulated by membrane tension that is determined by a combined contribution from osmotic pressure, in-plane tension, and cytoskeletal forces [48,49]. The forces that are transmitted from the cell membrane to the nucleus maintain and regulate nuclear functions, including gene expression [50]. In fact, membrane tension and cell volume are coupled during osmosis, which is actively regulated by ion transporters, mTOR pathways, and cytoskeleton [35], and changes in cell volume size always concur with drastic cytoskeleton reorganizations [51]. In this study, we observed clear perinuclear translocation of actin cytoskeleton together with increased membrane tension in the ASMCs when cell volume was increased. All these effects of cell volume on contractile function and cytoskeleton organization of the ASMCs could be prevented or reversed if the cell membrane tension was reduced by pretreating the cells with membrane tension inhibitors such as Ble, Y27632, and ML-7. Therefore, our findings indicate that membrane tension is required for maintaining the cell volume-enhanced contractile function associated with ASMC hypertrophy.

On the other hand, the membrane tension is sensed by the mechanosensitive channel Piezo1 to convert extracellular mechanical signals into intracellular biochemical signals to control cell function. Our study found that Piezo1 was regulated by cell volume at least at the level of mRNA expression, which, in turn, played a key role in the cell volume-induced contractile function. Other studies have revealed some similar effects of Piezo1 on cell behaviors. For example, Piezo1 activation has been related to increased cell volume, rigidity, and contractile forces in vascular smooth muscle cells [52]. Downregulation of Piezo1 in prostate cancer cells cultured in vitro could inhibit the cells’ migration [53]. In our previous study, we also found that Piezo1 could be chemically activated to alter the mechanical behaviors of ASMCs, such as migration and relaxation, that are primarily associated with pathogenic airway remodeling [54]. In addition, stretch could activate Piezo1 to lead to potential downstream changes in contractility and migration of ASMCs and ECM remodeling [55,56]. These together suggest that Piezo1 may have great potential to be a novel target for ameliorating ASMC hypertrophy in a disease [57,58].

Because YAP is an important downstream protein of Piezo1, it should also be involved in the regulation of cell behavior due to cell volume change [59,60]. Multiple studies have evidenced the involvement of YAP in promoting ASMC proliferation and migration during the progression of airway remolding [61,62,63]. In this study, we demonstrated that the mRNA expression and perinuclear localization of YAP were all correlated with the cell volume of the ASMCs, and the cell volume-induced YAP mRNA expression and translocation could be largely reduced by either lowering membrane tension or knocking down Piezo1 expression. On the other hand, knocking down YAP expression largely attenuated the contractile function of hypertrophic ASMCs, including the contractile protein expression, baseline cell stiffness, and ACh-induced cell stiffness response, as well as the mRNA expression of Piezo1. These findings seem to reveal an auto-regulatory loop between Piezo1 and YAP that is involved in the contractile function regulation during ASMC hypertrophy.

It is important to note that, apart from expression and activation, the intracellular distribution of Piezo1 is also highly dynamic at the single-cell level. For example, studies have shown that Piezo1 tended to enrich at certain subcellular locations, such as focal adhesion sites and nuclei, and moved in clusters from the cell soma to the extrusion edges during cell expansion, which required myosin II contractility [64,65,66,67]. In our model, Piezo1 appeared to be internalized via caveolin-2-dependent endocytosis. These studies seem to suggest that Piezo1 function probably depends critically on various physical states of the cells, including size, behavior, and force application.

Our study is relatively limited in the revelation of the detailed mechanisms of contractile function regulation during cellular hypertrophy. At least the roles of Piezo1 and YAP and their crosstalk in the regulation of contractile function in response to cell volume change need to be investigated at protein and transcription levels in future studies. Moreover, the cultured ASMCs pretreated with Piezo1 negative control siRNA and their normal non-transfected counterparts appeared somewhat different in response to ACh stimulation. This suggests that the transfection reagents may cause damage to cells and, thus, affect the observed contractile function of the cells. In addition, complementary in vivo experimental data should be considered to confirm the role of the Piezo1/YAP axis in promoting the tension function of hypertrophic ASMCs.

## 5. Conclusions

We successfully established an in vitro model to simulate ASMC hypertrophy at the single-cell level and, thereby, studied the effects and regulatory mechanism of cell volume on the cell’s contractile function. We demonstrated that cell volume increase led to enhanced contractile function, which was facilitated through cytoskeleton reorganization and membrane tension modulation. More importantly, membrane tension-regulated Piezo1, together with an auto-regulatory loop between Piezo1 and YAP mRNA expression, was required for the enhanced contractile function due to cell volume increase in the ASMCs. Taken together, the present study provides evidence that ASMC hypertrophy can be directly linked to the enhanced contractile function of ASMCs and, thus, to the airway hyperresponsiveness and the essential role of Piezo1 in mediating this link, which may help not only better understand the pathogenic mechanism but also explore a specific therapeutic target for preventing/treating airway diseases associated with the ASMC hypertrophy such as severe asthma.

## Data Availability

The raw data supporting the conclusions of this article will be made available by the authors on request.

## Supplementary Materials

The following supporting information can be downloaded at: https://www.mdpi.com/article/10.3390/cells13201697/s1, Table S1: Primers used for real-time PCR analysis; Figure S1: Characteristics of ASMCs cultured in low density; Figure S2: Micropatterning to control ASMC length; Figure S3: SMA and P-MLC expression; Figure S4: The area under the curve for each group; Figure S5: Baseline calcium fluorescence; Figure S6: The area and volume of the nucleus; Figure S7: Piezo1 internalization through caveolae dependent endocytosis; Figure S8: Piezo1 or YAP expression.

## Abbreviations

ACh, acetylcholine; AHR, hyperresponsiveness; AFM, atomic force microscope; ASMCs, airway smooth muscle cells; Ble, Blebbistatin; DMEM, Dulbecco’s modified Eagle’s medium; FBS, fetal bovine serum; Fluo-4/AM, Fluo-4 acetoxymethylester; FN, fibronectin; FTTFM, Fourier transformation traction force microscopy; YAP, yes-associated protein; PBS, phosphate buffered saline; PDMS, polydimethylsiloxane; NC, negative control; KD, knockdown; P-MLC, phosphorylated myosin light chain 2; SMA, smooth muscle α-actin; SMMHC, smooth muscle myosin heavy chain; OMTC, optical magnetic twisting cytometry; siRNA, small interfering RNA.
