# Supplementary material for: Single-Cell Hypertrophy Promotes Contractile Function of Cultured Human Airway Smooth Muscle Cells via Piezo1 and YAP Auto-Regulation
Source: Cells. 2024 Oct 14;13(20):1697. doi: 10.3390/cells13201697 (PMC11505810; doi:10.3390/cells13201697)
Supplement: Supplementary file 1 [file cells-13-01697-s001.zip › cells-3181402-supplementary.pdf]

Single-cell hypertrophy promotes contractile function of cultured human airway smooth muscle cells via Piezo1 and YAP auto-regulation

Kai Ni [†], Bo Che [†], Rong Gu, Chunhong Wang, Yan Pan, Jingjing Li, Lei Liu, Mingzhi Luo *, and Linhong Deng *

Changzhou Key Laboratory of Respiratory Medical Engineering, Institute of Biomedical Engineering and Health Sciences, School of Medical and Health Engineering, Changzhou University, Changzhou 213164, China

[†] These Authors contributed equally

* Correspondence: luomingzhi@cczu.edu.cn (L.M.), dlh@cczu.edu.cn (L.D.)

Supplementary Table S1. Primers used for real-time PCR analysis.

Supplementary Figure S1. Characteristics of ASMCs cultured in low density.

Supplementary Figure S2. Micropatterning to control ASMC length.

Supplementary Figure S3. SMA and P-MLC expression.

Supplementary Figure S4. The area under the curve for each group.

Supplementary Figure S5. Baseline calcium fluorescence.

Supplementary Figure S6. The area and volume of the nucleus.

Supplementary Figure S7. Piezo1 internalization through caveolae dependent endocytosis.

Supplementary Figure S8. Piezo1 or YAP expression.

Table S1 Primers used for real-time PCR analysis

Gene	Sequence of primers
calponin	Forward: 5’-AGGTGAACGTGGGAGTGAAG-3’
	Reverse: 5’-GGCTGGCAAACTTGTTGGTG-3’)
SMA	Forward: 5’-CACAAGTATCACGGGAGAGC-3’
	Reverse: 5’-TTGTCGTTCTTCACTGTTTTGG-3’
SMMHC	Forward: 5’- GCAAGAAGAGGCACGAGATG-3’
	Reverse: 5’- ACGGCCAGGTACTGAATGAC-3’
Piezo1	Forward: 5’- GAGGATGTTCAACCAGAATG-3’
	Reverse: 5’- GGAAGAGGTTGAGATGATTG-3’
YAP	Forward: 5’- TAGCCCTGCGTAGCCAGTTA-3’
	Reverse: 5’- TCATGCTTAGTCCACTGTCTGT-3’
E2F7	Forward: 5’-CACACACGTTAAACACCAACCT-3’
	Reverse: 5’- CGTGTGGGGCACGTGGCAAC-3’
GADPH	Forward: 5’-TGACGCTGGGGCTGGCATTG-3’
	Reverse: 5’-GGCTGGTGGTCCAGGGGTCT-3’

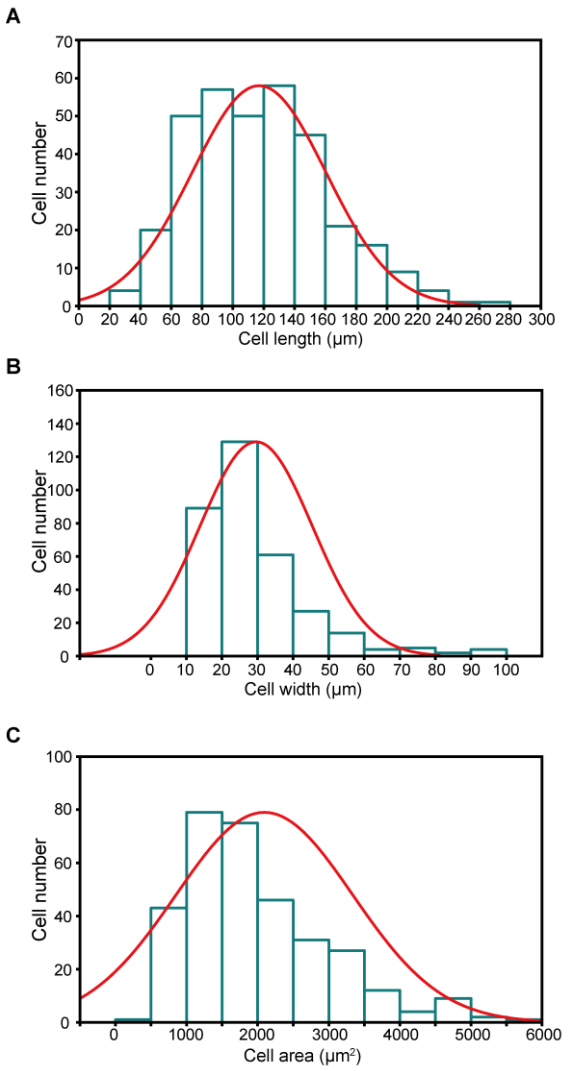

Figure S1. Characteristics of ASMCs cultured in low density on the petri dish. (**A–C**) Cell number histogram of cell size (length, width, and area) in ASMCs cultured in low density on the petri dish.

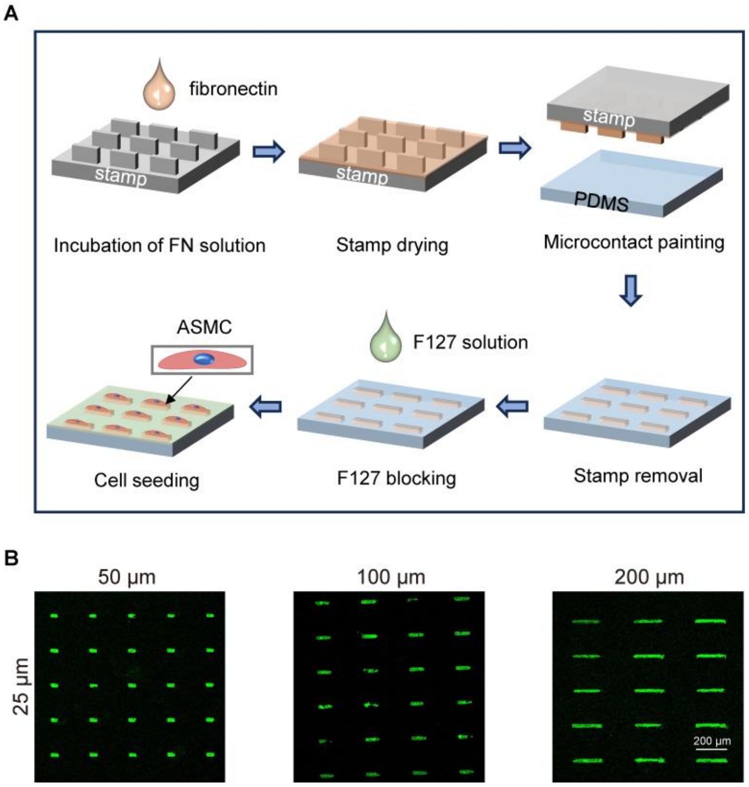

Figure S2. Micropatterning to control ASMC length. (**A**) The flow diagram is used to prepare the substrate with micropatterns (FN: fibronectin). (**B**) Representative image of rectangular patterns of different lengths (50, 100, and 200 μm), with an equal width (25 μm) coated with FITC-fibronectin. Scale bar = 200 μm.

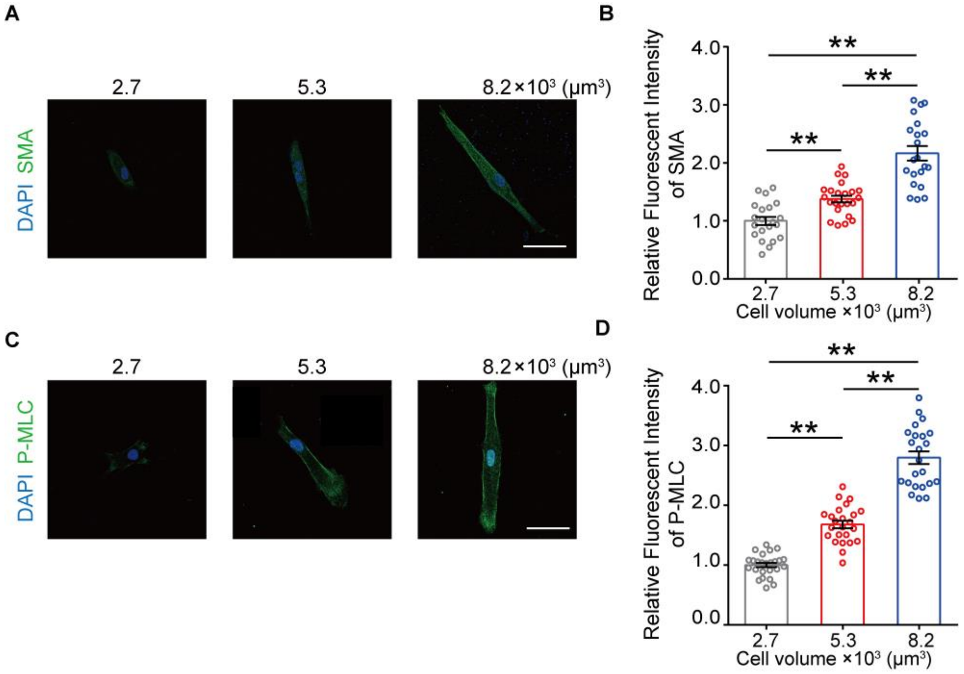

Figure S3. SMA and P-MLC expression. Representative images **(A)** and quantified fluorescent intensity **(B)** of ASMCs with immunofluorescence staining (green: SMA, blue: DAPI for nucleus) versus cell volume (n = 18–21 cells). Representative images **(C)** and quantified fluorescent intensity **(D)** of ASMCs with immunofluorescence staining (green: P-MLC, blue: DAPI for nucleus) versus cell volume (n = 24–26 cells). Scale bar = 50 μm.

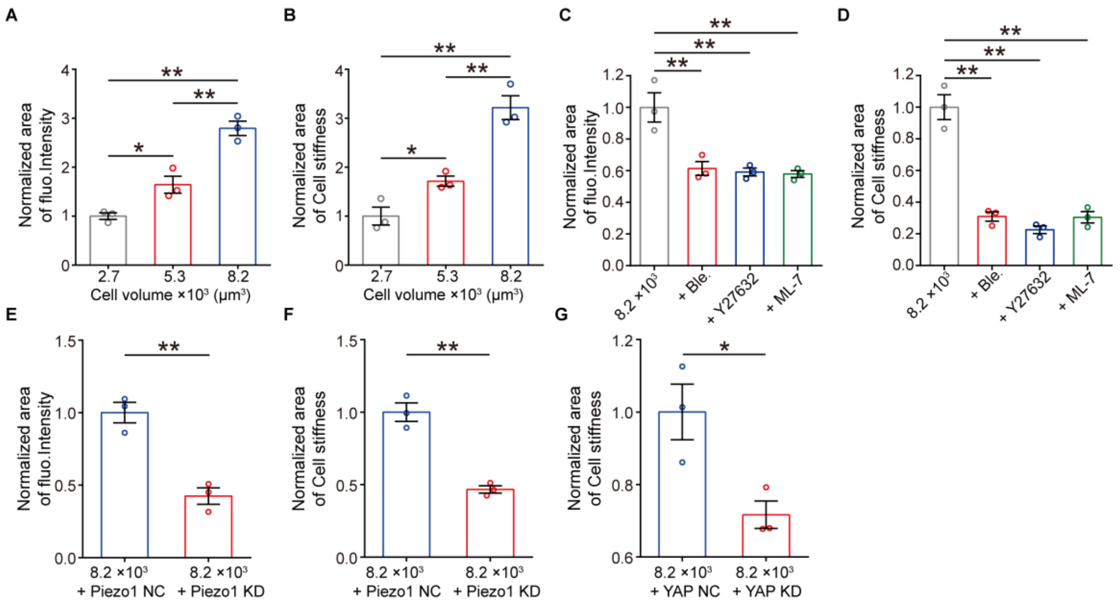

Figure S4. The area under the curve for each group. Quantification of the normalized area under the curve for the released calcium **(A)** and the stiffness **(B)** induced by ACh with different volumes (n = 3). Quantification of the normalized area under the curve for the released calcium **(C)** and the stiffness **(D)** induced by ACh with cell volume of 8200 μm^3 after the treatment with vehicle, Ble, Y-27632, or ML-7 (n = 3). Quantification of the normalized area under the curve for the released calcium **(E)** and the stiffness **(F)** induced by ACh with cell volume of 8200 μm^3 under Piezo1 KD or NC (n = 3). Quantification of the normalized area under the curve for the stiffness **(G)** induced by ACh with cell volume of 8200 μm^3 under YAP KD or NC (n = 3).

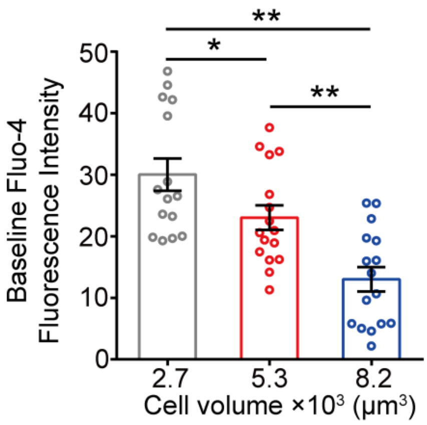

Figure S5. Baseline calcium fluorescence. Quantification of the baseline calcium fluorescence of the ASMCs versus the cell volume (n = 15–20 cells).

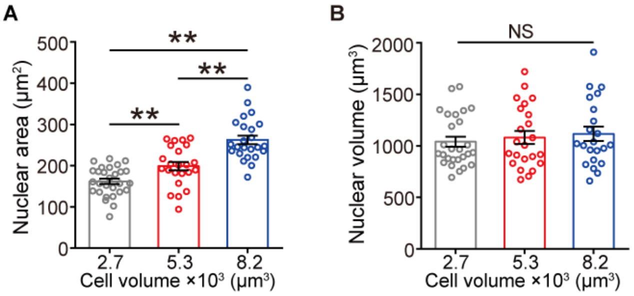

Figure S6. The area and volume of the nucleus. Quantification of the area (**A**) and volume (**B**) of the ASMCs nucleus versus the cell volume (n = 20–28 cells).

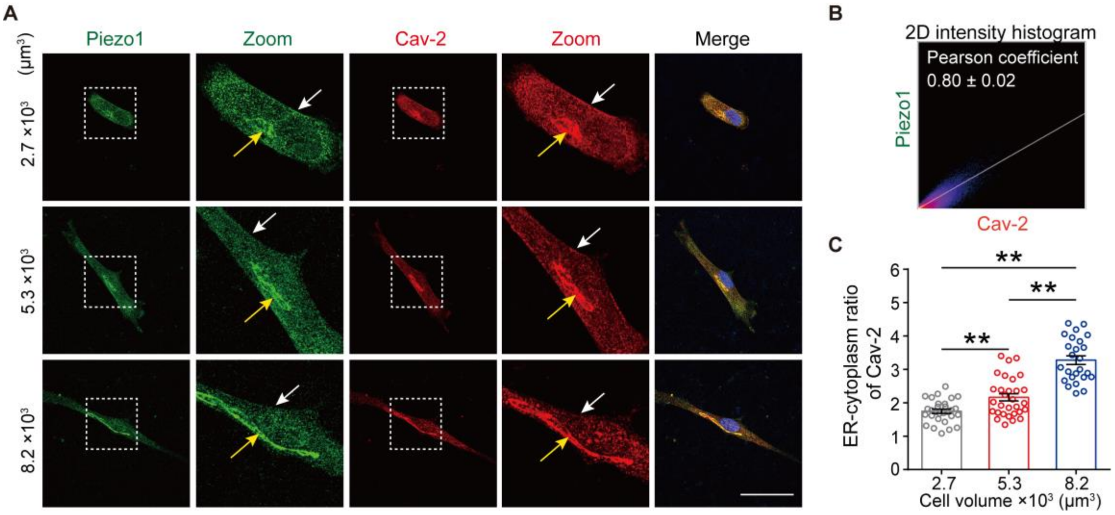

Figure S7. Piezo1 internalization through caveolae dependent endocytosis. (**A**) Representative fluorescence images of Piezo1 (green) and caveolae-2 (Cav-2, red) colocalization in ASMCs with different cell volumes (2.7, 5.3, and 8.2 $\times 10^3$ μm^3, respectively). (**B**) Representative image of 2D intensity histogram output of Coloc2 analysis performed using Fiji software. The text indicates the Pearson coefficient of the pixel-intensity correlation (n = 8). (**C**) Quantified the ratio of the endoplasmic reticulum (ER) to cytoplasm of Cav-2 fluorescent intensity of in ASMCs with different cell volumes (n = 27–30 cells). Data are means ± S.E.M. Scale bar = 50 μm. ** $p < 0.01$.

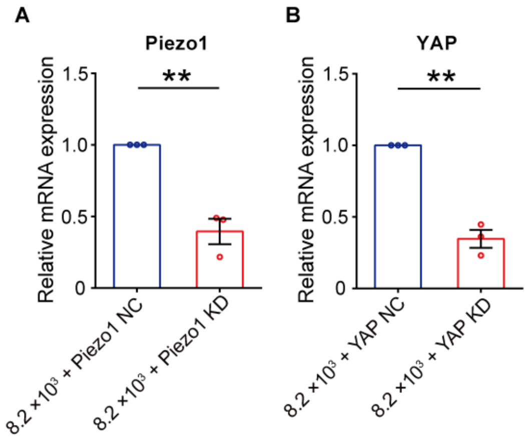

Figure S8. Piezo1 or YAP expression. (**A**) Piezo1 mRNA expression in the hypertrophic ASMCs (8.2 $\times 10^3$ μm^3) pretreated with either Piezo1 NC or KD siRNA. (**B**) YAP mRNA expression in the hypertrophic ASMCs (8.2 $\times 10^3$ μm^3) pretreated with either YAP NC or KD siRNA. n = 3. ** $p < 0.01$.
